# Superior Measurement Accuracy of Digital Thickness Gauge Versus Digital Vernier Caliper in Determining Venous Tissue Thickness

**DOI:** 10.7759/cureus.68442

**Published:** 2024-09-02

**Authors:** Alexandru Petru Ion, Alexandra Asztalos, Claudiu Constantin Ciucanu, Eliza Russu, Adrian Vasile Muresan, Eliza-Mihaela Arbănași, Réka Bartus, Carmen Corina Radu, Traian V Chirilă, Emil-Marian Arbănași

**Affiliations:** 1 Regenerative Medicine Laboratory, Centre for Advanced Medical and Pharmaceutical Research (CCAMF), George Emil Palade University of Medicine, Pharmacy, Science, and Technology of Targu Mures, Targu Mures, ROU; 2 Department of General Medicine, George Emil Palade University of Medicine, Pharmacy, Science, and Technology of Targu Mures, Targu Mures, ROU; 3 Department of Vascular Surgery, George Emil Palade University of Medicine, Pharmacy, Science, and Technology of Targu Mures, Targu Mures, ROU; 4 Doctoral School of Medicine and Pharmacy, George Emil Palade University of Medicine, Pharmacy, Science, and Technology of Targu Mures, Targu Mures, ROU; 5 Department of Vascular Surgery, Mures County Emergency Hospital, Targu Mures, ROU; 6 Department of Forensic Medicine, George Emil Palade University of Medicine, Pharmacy, Science, and Technology of Targu Mures, Targu Mures, ROU; 7 Department of Scientific Research, Queensland Eye Institute, Queensland, AUS; 8 Department of Scientific Research, School of Chemistry and Physics, Queensland University of Technology, Queensland, AUS; 9 Department of Scientific Research, Australian Institute for Bioengineering and Nanotechnology (AIBN) University of Queensland, Queensland, AUS; 10 Faculty of Medicine, George Emil Palade University of Medicine, Pharmacy, Science, and Technology of Targu Mures, Targu Mures, ROU

**Keywords:** specimen thickness, biomechanics, venous tissue, digital vernier caliper, digital thickness gauge

## Abstract

Background

In determining mechanical characteristics, the accuracy of the thickness of the specimens can influence the biomechanical behavior, especially in the case of human tissues, where there is an important variability. This study aims to compare the accuracy of two routine measuring instruments, i.e., the digital vernier caliper and the digital thickness gauge, when measuring the thickness of venous specimens multiple times.

Methodology

In this study, we used 12 tubular vena cava specimens obtained from common breed pigs aged 18-24 weeks at the time of sacrifice from a local slaughterhouse. The measurements were performed using a digital vernier caliper (Multicomp PRO MP012475) for the first four protocols and a digital thickness gauge (Mitutoyo 547-500S) for the fifth protocol. In the first protocol, three measurements were taken on the same side, and their average was recorded as the sample thickness. The second protocol involved taking measurements on two opposite sides, and the average of these measurements was recorded as the sample thickness. In the third protocol, the thickness of each side was measured at its midpoint, and the average of the four measurements was recorded as the sample thickness. In the last protocol using a digital vernier caliper, the thickness of the vernier specimens was calculated as the average of the measurements taken at each corner of the square sample. Finally, for the fifth protocol, three consecutive measurements were taken using the digital thickness gauge, and their average was recorded as the final thickness of the sample.

Results

In the first protocol, we observed lower values during the first measurement in comparison to the second (0.409 ± 0.063 vs. 0.536 ± 0.064, p < 0.0001) and the third (0.409 ± 0.063 vs. 0.528 ± 0.055, p = 0.0001). Moreover, with the second protocol, we observed lower values during the first two measurements in comparison to the third measurement (p = 0.0279 and p = 0.0054). Regarding protocols three and four, we recorded higher values for the second and third measurements than the first one, with higher values for the third measurement than the second one. In the fifth protocol, there were no significant statistical differences between the three consecutive measurements (p = 0.953, p = 0.742, and p = 0.897). Further, we examined the variations in sample thickness determined using each of the protocols proposed for the digital vernier caliper, as well as the values obtained with the digital thickness gauge protocol. As a result, during the first and second measurements, we observed lower thickness values for the venous wall samples using the first four protocols compared to the fifth protocol (for all p < 0.05). However, no differences were noted between the five protocols during the third measurement.

Conclusions

The digital thickness gauge Mitutoyo 547-500S provided superior accuracy with no difference between three successive measurements of venous wall thickness, regardless of the examiner’s experience. Accurately determining the thickness of venous specimens is crucial for calculating the tissue’s biomechanical properties.

## Introduction

Autologous vein grafts are widely used in various revascularization surgical procedures and are recommended as the first choice for aorto-coronary bypass and lower limb revascularization [1,2]. However, the failure rate of vein grafts is approximately 30% at five years and can reach up to 60% at 10 years [3]. The primary cause of venous graft failure is intimal hyperplasia, resulting from endothelial injury due to arterial pressure and new hemodynamic changes [4-7].

Recently, biomechanical analysis of vascular tissue has provided valuable information regarding the behavior and remodeling of arterial and venous grafts [8-10]. The noteworthy elements are the elasticity and resistance to stretching of the tissues, determined mathematically by Cauchy stress and Young’s modulus [11,12].

In determining the mechanical characteristics, the accuracy of the thickness of the specimens can influence the biomechanical behavior, especially in the case of human tissues, where there is an important variability [13]. Therefore, different measurement techniques and various measuring devices can be used. The most used instruments today are digital vernier caliper, micrometer, and thickness gauge digital caliper [14].

This study aims to compare the accuracy of two routine measuring instruments, i.e., the digital vernier caliper and the digital thickness gauge, when measuring the thickness of venous specimens multiple times. Additionally, we compare five protocols, four for the digital vernier caliper and one for the thickness gauge digital caliper, to determine which protocols demonstrate the best reproducibility.

## Materials and methods

Sample preparation

In this study, we used 12 tubular vena cava specimens obtained from common breed pigs aged 18-24 weeks at the time of sacrifice from a local slaughterhouse. The specimens were immediately transported to the laboratory after harvesting, where 12 samples measuring 10 × 10 mm were prepared and stored in phosphate-buffered saline for further thickness measurement. The pigs were slaughtered for commercial purposes, and the vascular tissue used in our study would have been discarded if not utilized. This study was approved by the Committee of Ethics in Scientific Research of the George Emil Palade University of Medicine, Pharmacy, Science, and Technology of Targu Mures, Romania (protocol number: 2653/15.12.2023).

Measurements protocols

**Figure 1:**
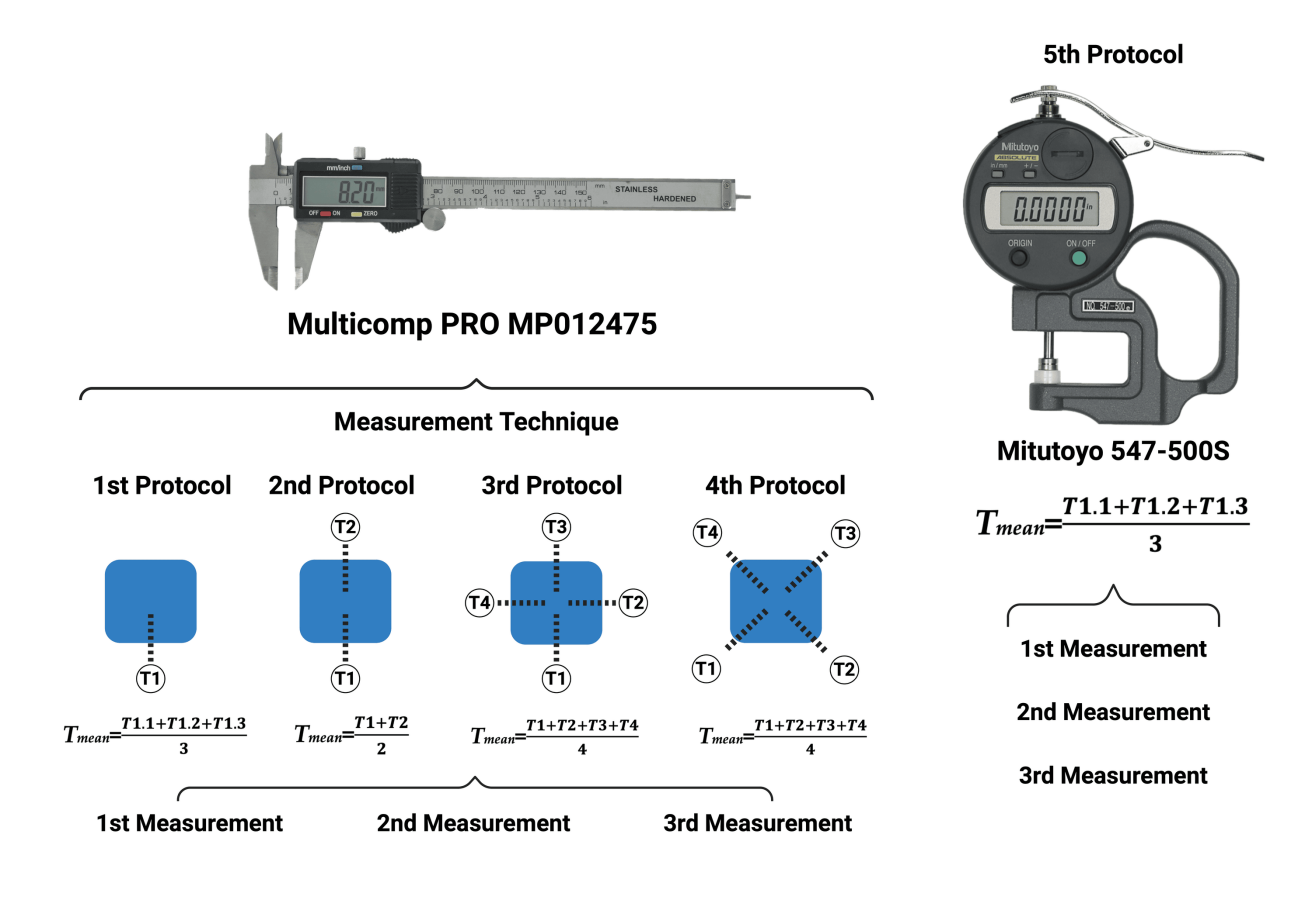
Graphic representation of the five protocols used in this study for determining the thickness of porcine vena cava specimens.

The measurements were performed using a digital vernier caliper (Multicomp PRO MP012475) for the first four protocols and a digital thickness gauge (Mitutoyo 547-500S) for the fifth protocol (Figure 1). In the first protocol, three measurements were taken on the same side, and their average was recorded as the sample thickness. The second protocol involved taking measurements on two opposite sides, and the average of these measurements was recorded as the sample thickness. In the third protocol, the thickness of each side was measured at its midpoint, and the average of the four measurements was recorded as the sample thickness. In the last protocol using a digital vernier caliper, the thickness of the vernier specimens was calculated as the average of the measurements taken at each corner of the square sample. Finally, in the fifth protocol, three consecutive measurements were taken using the digital thickness gauge, and their average was recorded as the final thickness of the sample. All determinations were performed by the same individual to eliminate variability bias among users.

Study outcome

The primary objective was to assess the consistency and accuracy of the porcine cava vein specimen thickness by comparing measurements taken using a digital vernier caliper and a digital thickness gauge.

Statistical analysis

The statistical analysis was performed using SPSS software for MacOS version 29.0.1.1 112 (IBM Corp., NY, USA, USA). The values included in the analysis were the averages of the dimensions obtained after each measurement. Values are represented as the average value and standard deviation and the median (quartile 1-quartile 3). The Mann-Whitney U test was used to compare the means and evaluate the differences between ratios among the four protocols and the control protocol (the fifth). We considered a p-value of less than 0.05 as statistically significant.

## Results

**Figure 2:**
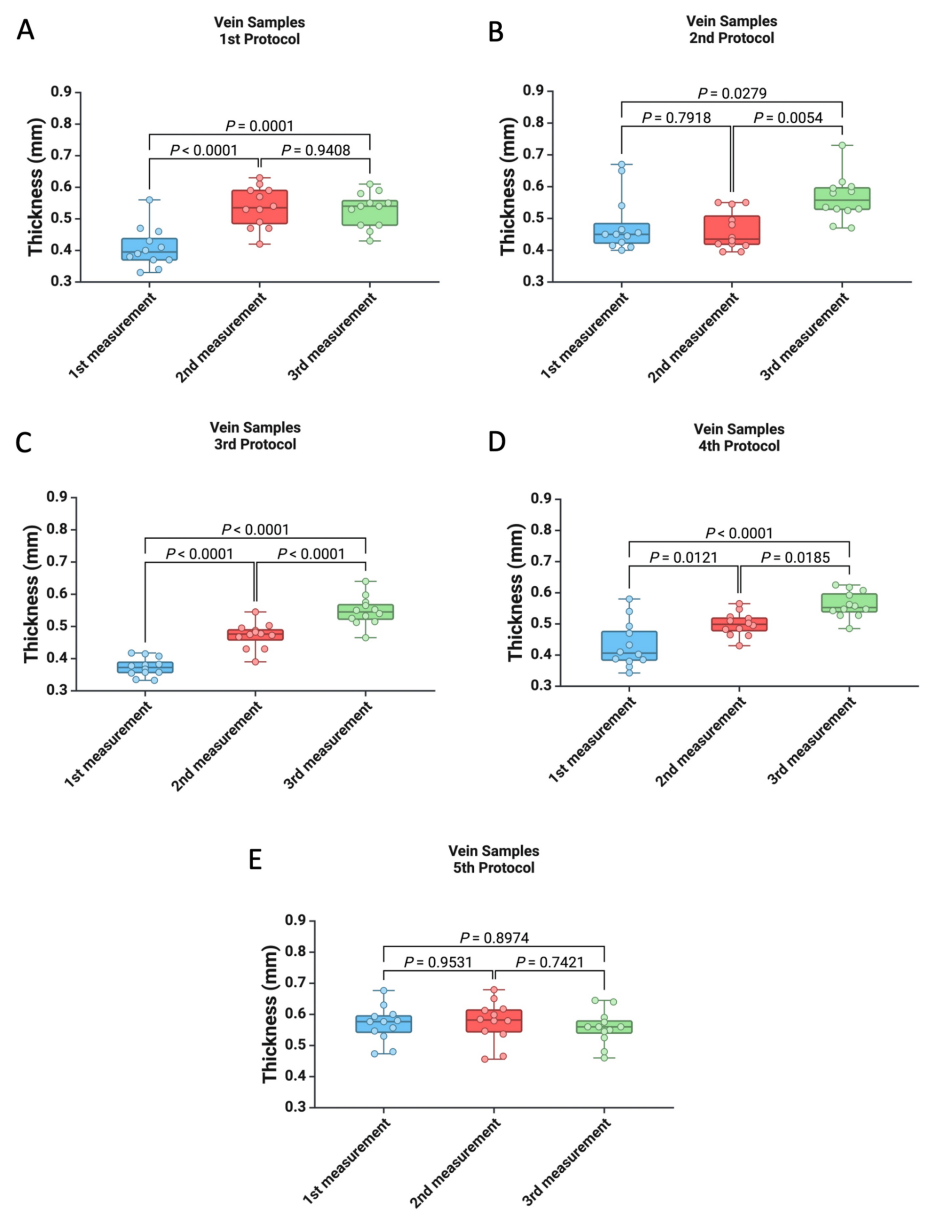
Graphic representation of the differences between consecutive measurements of specimens for each protocol: (A) first protocol, (B) second protocol, (C) third protocol, (D) fourth protocol, and (D) fifth protocol. A p-value of less than 0.05 was considered statistically significant and was analyzed using the Mann-Whitney U test.

In this study, we determined the thickness of 12 porcine venous wall specimens using five different protocols, four of which used a digital vernier caliper and one of which used a digital thickness gauge. As shown in Figure 2 and Table 1, of the five protocols used, only in the fifth protocol there were no significant statistical differences registered between the three consecutive measurements (p = 0.953, p = 0.742, and p = 0.897 ). In the first protocol, we observed lower values during the first measurement in comparison to the second (0.409 ± 0.063 vs. 0.536 ± 0.064, p < 0.0001) and the third (0.409 ± 0.063 vs. 0.528 ± 0.055, p = 0.0001). Moreover, in the second protocol, we observed lower values during the first two measurements in comparison to the third measurement (p = 0.0279 and p = 0.0054). Regarding protocols three and four, we recorded higher values for the second and third measurements than the first one, with higher values for the third measurement than the second one (Figure 2, Table 1).

**Table 1:** Thickness of vein wall specimens for the three consecutive measurements with the five protocols.

	First measurement	Second measurement	Third measurement
First protocol – digital Vernier caliper			
Mean ± SD (mm)	0.409 ± 0.063	0.536 ± 0.064	0.528 ± 0.055
Median (Q1-Q3) (mm)	0.395 (0.370-0.452)	0.535 (0.475-0.590)	0.540 (0.480-0.572)
Second protocol – digital vernier caliper			
Mean ± SD (mm)	0.481 ± 0.091	0.461 ± 0.061	0.562 ± 0.071
Median (Q1-Q3) (mm)	0.450 (0.417-0.521)	0.435 (0.416-0.532)	0.557 (0.526-0.598)
Third protocol – digital vernier caliper			
Mean ± SD (mm)	0.373 ± 0.028	0.471 ± 0.039	0.547 ± 0.044
Median (Q1-Q3) (mm)	0.372 (0.355-0.401)	0.476 (0.439-0.493)	0.545 (0.517-0.572)
Fourth protocol – digital vernier caliper			
Mean ± SD (mm)	0.432 ± 0.073	0.498 ± 0.037	0.561 ± 0.041
Median (Q1-Q3) (mm)	0.406 (0.381-0.486)	0.498 (0.469-0.521)	0.552 (0.531-0.604)
Fifth protocol – digital thickness gauge			
Mean ± SD (mm)	0.568 ± 0.057	0.575 ± 0.066	0.557 ± 0.055
Median (Q1-Q3) (mm)	0.576 (0.532-0.598)	0.582 (0.539-0.616)	0.560 (0.530-0.586)

**Figure 3:**
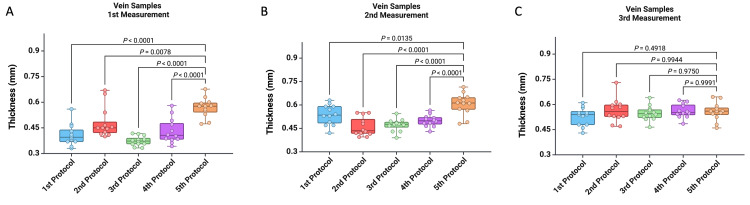
Graphic representation of the differences between the first four protocols and the fifth protocol for the (A) first measurement, (B) second measurement, and (C) third measurement. A p-value of less than 0.05 was considered statistically significant and was analyzed using the Mann-Whitney U test.

Further, we examined the variations in sample thickness determined using each of the protocols proposed for the digital vernier caliper, as well as the values obtained with the digital thickness gauge protocol. As a result, during the first and second measurements, we observed lower thickness values for the venous wall samples using the first four protocols compared to the fifth protocol (Figure 3). However, no differences were noted between the five protocols during the third measurement (Figure 3).

**Figure 4:**
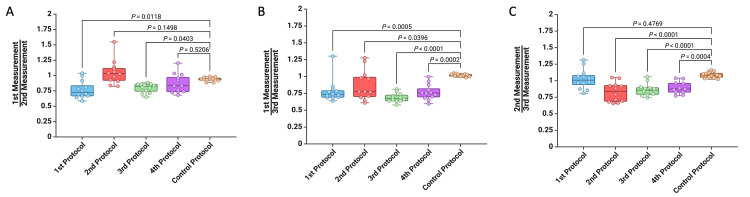
Graphic representation of the differences between the first four protocols and the control protocol (fifth Protocol) for (A) the ratio between the first and second measurement, (B) the ratio between the first and third measurement, and (C) the ratio between the second and third measurement. A p-value of less than 0.05 was considered statistically significant and was analyzed using the Mann-Whitney U test.

In addition, as there were no significant statistical differences in the fifth protocol over three consecutive determinations, we used it as a control to assess the accuracy of the first four protocols. We then calculated the ratios between the values obtained in the first, second, and third measurements (Figure 4). When comparing the ratio between the values determined at the first and second measurements, we found no significant statistical differences between the second and fourth protocols and the control protocol (p = 0.1498 and p = 0.5206) (Figure 4A). Similarly, when comparing the ratio between the second and third measurements, we found no differences between the first protocol and the control protocol (p = 0.4769) (Figure 4C). Furthermore, we observed smaller differences between the other protocols and the control protocol.

## Discussion

This study’s main finding is that the digital thickness gauge Mitutoyo 547-500S provided superior accuracy with no difference between three successive measurements of venous wall thickness, regardless of the examiner’s experience. In contrast, significant differences were found between the three measurements in all four protocols when using the digital vernier caliper. Moreover, we observed higher venous wall thickness values in the fifth protocol compared to the first four protocols for the first two measurements, with no difference between the protocols for the third measurement.

It has been demonstrated that other measurement methods can be used, such as the micrometer, glass slide, and laser, in addition to those mentioned in our study, with the thickness gauge again showing its superiority over the rest of the methods, and vernier caliper not having significant reliability and being dependent on the experience of the evaluator [14]. Interest in establishing a more accurate method for determining tissue thickness was expressed as early as 1996, when Lee et al. compared five methods of determining bovine pericardial thickness, namely, a Mitutoyo non-rotating thickness gauge, a custom-built instrumented thickness gauge that was strain gauged to measure contact force, a commercial Hall effect probe (Panametrics Magna-Mike), a custom-built electrical resistance probe, and measurement of fresh frozen histological sections under polarized light. They reported that the first method represented the most pragmatic choice in this regard [15].

Determining the thickness of the analyzed sample as precisely as possible is crucial for the biomechanical analysis of the tissues and their correct interpretation. This translates into obtaining the most optimal result when the vein tissue is used for aortocoronary bypass or revascularization of the lower limb in patients with peripheral arterial disease [16-18]. It must also be taken into account that the vein tissue used for the procedure will undergo a hyperplasia process following its exposure to the mechanical stress exerted by the arterial flow [16,19]. For this reason, the use of veins with an altered structure of the wall should be avoided, although in some cases, similar results have been highlighted in terms of graft patency [20]. Moreover, this study has implications for other kinds of tissues, including muscles, nerves, and arteries, alongside veins, that undergo biomechanical analysis [11,12]. Studying the biomechanical behavior of human venous tissue can help us better understand how the tissue remodels and how regular factors affect the histological structure of the venous wall [21-29]. This understanding can aid in developing new devices and stents [26-29]. In a study by Hamadeni et al. [26], the great saphenous vein (GSV) exhibited higher stiffness in the longitudinal axis (5.38 ± 0.6 vs. 1.65 ± 0.11 MPa) and the circumferential axis (2.61 ± 0.67 vs. 0.97 ± 0.19 MPa) compared to the umbilical vein wall. Karimi et al. [27] found that the umbilical artery wall is stiffer than the umbilical vein wall (p < 0.05), although there were no differences in terms of maximum stress. Currently, there is a focus on developing tissue-engineered biological grafts with biomechanical properties similar to natural arteries. Tosun et al. [28] showed that early biomechanical changes can be an essential biomarker for long-term adverse events in tissue-engineered grafts. Additionally, Vesely et al. [29] analyzed the constitutive modeling of the GSV at different pressures and demonstrated that at an angle of 40 degrees, the flow pressure is uniformly distributed over the entire wall thickness.

Limitations

This study is the first of its kind, paving the way for further investigation of venous tissue. However, our study has some limitations. The first is the measurement of the samples by a single examiner, which, even though removes the bias of the examinations from several examiners, brings with it the risk of the examiner getting acquainted with the vernier caliper, as can be seen throughout the three measurements. Even though our results demonstrate the superiority of the digital thickness gauge Mitutoyo 547-500S, it is important to note that the person who measured the specimen thickness lacks experience with the instruments. It would be interesting to investigate in the future whether an experienced user can accurately determine the thickness of the specimens using the two types of instruments without finding significant statistical differences between the values. In addition to this, the impact of the thickness of the samples on their biomechanical properties should be evaluated for a better understanding of the behavior of the venous tissue.

## Conclusions

The digital thickness gauge Mitutoyo 547-500S consistently provided highly accurate measurements of venous wall thickness, regardless of the examiner’s experience level. Conversely, using the digital vernier caliper resulted in significant differences between consecutive measurements for all four protocols utilized in this study. Moreover, using a digital thickness gauge to measure venous wall thickness took less time than using a digital vernier caliper, and the results were not affected by the examiner’s accuracy. Accurate thickness determination and biomechanical analysis of vascular tissue are essential for developing new stents and stent grafts for various vascular conditions, including peripheral arterial disease, aortic aneurysmal disease, and coronary and carotid atherosclerotic disease. Furthermore, these insights are crucial for the development of new devices and therapeutic strategies aimed at enhancing the quality of life for vascular patients.
